# Low Temperature Transient Liquid Phase Bonding of Alumina Ceramics with the Bi_2_O_3_-ZnO Interlayer

**DOI:** 10.3390/ma15196940

**Published:** 2022-10-06

**Authors:** Maria Stosz, Sathya Narayanasamy, Thomas Graule, Dariusz Kata, Gurdial Blugan

**Affiliations:** 1 High Performance Ceramics Laboratory, Swiss Federal Laboratories for Materials Science and Technology (Empa), Ueberlandstrasse 129, 8600 Dübendorf, Switzerland; 2 Faculty of Materials Science and Ceramics, AGH University of Science and Technology, Al. Mickiewicza 30, 30-059 Cracow, Poland

**Keywords:** joining, interlayer, bonding, transient-liquid-phase bonding, joining of ceramics, low-temperature joining

## Abstract

Alumina ceramics were joined by a transient liquid phase (TLP) bonding method at relatively lower temperatures, using mixed powders of Bi_2_O_3_ and ZnO with different weight ratios as interlayers between the ceramic components. Bonding was achieved at 750 °C for several of the prepared interlayer mixtures, which makes the applied approach attractive due to the relatively lower joining temperature and potentially low fabrication costs. Measurements by SEM and EDX were used to study the microstructure and chemical analysis of the obtained joints. It also allowed us to investigate the diffusion mechanism occurring in the systems, which resulted in the hypothesis that Zn^2+^/ZnO diffuses through the ceramics. XRD and Raman spectra were acquired to examine the reaction products that formed during the thermal treatment. The results showed that both ZnO and Bi_2_O_3_ react with each other as well as with alumina to form spinel and other products.

## 1. Introduction

Ceramic materials are known for their very specific properties. They usually have good durability, and they are often thermally resistant [1,2,3,4]. However, in many cases, the manufacturing of ceramics is challenging. It requires using high processing temperatures and it consists of several stages, which additionally makes the fabrication a time-consuming process. Hence, the whole process generates significant costs and leads to complications. Due to the application of multiple steps during the manufacturing process, defects like cracks and pores are extremely hard to avoid. Furthermore, preparing parts with a complicated shape is difficult [5,6]. Therefore, it is very important to develop new techniques for manufacturing ceramic materials to make the process more affordable. One of the approaches involves creating simpler and smaller components at first and then joining them together by using an interlayer made of a similar/different material. The joining processes are carried out at a higher temperature and often under high pressure [7,8]. The transient liquid phase (TLP) bonding method is nowadays gaining more and more attention among brazing and diffusion bonding techniques [9,10,11,12]. TLP owes its popularity to the lower process temperatures and generally higher fracture strengths of the obtained joints [13,14,15].

TLP bonding is also known as diffusion brazing or isothermal solidification [16]. An interlayer is inserted between the two substrates that need to be joined. During the joining process, a thin liquid phase forms at the contact point between the ceramic surface and the interlayer. Similar to brazing, the ability to wet the ceramic components with the liquid composition should be significant [17,18,19]. There are two main possible paths to achieve the liquid phase during joining. In the first one, the joining temperature is in the same range as the melting point of the interlayer material. The second approach involves the formation of eutectic products between the interlayer and the base material, typically at a temperature that is lower than the melting point of the interlayer [20]. During the TLP bonding process, the ceramic parts are joined until the liquid phase becomes isothermally solidified [21].

Alumina is used more frequently for research purposes as one of the most popular oxide ceramic material due to its thermal, electrical and mechanical properties [22,23]. It is widely used in many fields and finding new techniques for fabricating complicated parts in a simple and economical way is very important. Bulk alumina was previously joined by TLP bonding, mostly by applying metallic interlayers [24,25,26,27]. Among oxide interlayers, B_2_O_3_ and Bi_2_O_3_ were used to join ceramics at a relatively lower temperature [28,29]. B_2_O_3_ has a low melting point and forms compounds with alumina during joining at temperatures above 700 °C [28]. In this study, Bi_2_O_3_ was chosen for the low value of its melting point and proven reaction with alumina to form sillenite-type phases [29,30,31]. Joining of alumina ceramics with Bi_2_O_3_ was achieved in the literature at 850 °C and above [29]. However, this material has one disadvantage. Its linear coefficient of thermal expansion (CTE) is 14 × 10^−6^ K^−1^, which is significantly higher than that of alumina (around 7 × 10^−6^ K^−1^) [32]. This difference can create stresses during the cooling phase of the system consisting of alumina and Bi_2_O_3_ after thermal treatment, which can lead to a lower quality of bonding. To overcome this issue, ZnO was introduced to the Bi_2_O_3_ interlayer. The linear coefficient of thermal expansion of ZnO is 4.8 × 10^−6^ K^−1^ [33]. Therefore, it could be possible that the thermal stresses in a well-mixed Bi_2_O_3_-ZnO system with alumina could be lower than a system with only Bi_2_O_3_ and alumina. Moreover, it is known in the literature that ZnO reacts with alumina, forming a spinel phase [34,35]. It can be suggested that adding it to the reaction system could improve the strength of joints. Furthermore, its phase diagram with Bi_2_O_3_ shows that the addition of even a small quantity of ZnO reduces the temperature of the liquid phase formation in the Bi_2_O_3_-ZnO system to 738 °C [36]. Hence, the bonding process based on the TLP mechanism might be possible at a lower temperature, which is an added benefit for systems requiring lower assembly/joining temperatures due to other temperature-sensitive components.

## 2. Materials and Methods

Bonding experiments were performed using alumina discs (purity 99.7%, density 99.5%) with a diameter of 10 mm and a height of 1 mm. The linear coefficient of thermal expansion of the used material at room temperature is equal to 7.2 × 10^−6^ K^−1^. Their surfaces were cleaned ultrasonically for 15 min in acetone and in ethanol. Then they were dried in air. Bi_2_O_3_ powder with a purity of 99.995% and average size of 10 μm, and ZnO powder with a purity of 99.999% from Alfa Aesar by Thermo Fisher (Kendel, Germany) GmbH, were used as components of interlayers. Different compositions of powders, ranging from 0 to 30% weight of ZnO (Table 1), were mixed with acetone to form a slurry and then deposited on the alumina disks surfaces. The compositions were chosen based on the Bi_2_O_3_-ZnO phase diagram to examine most of the different zones present on it [36]. After the evaporation of the solvent, clean ceramic components were placed on top of the coated disks to form a sandwiched structure. Joining experiments were performed in the resistance furnace (PYROTEC Brennofenbau GmbH, Osnabrück, Germany) at temperatures ranging from 700 to 760 °C, with a heating and cooling rate equal to 60 °C/h. No additional pressure was applied, and the dwell time of experiments were either 2 or 3 h.

Bonded samples with a stronger durability were covered in resin and polished to an appropriate depth to examine the cross-sections of the joined ceramics. Measurements by SEM (Scanning Electron Microscopy) and EDX (Energy Dispersive X-ray) techniques (Tescan Vega 3 SEM microscope, Germany) were used to perform the microstructure and chemical analysis. SEM analyses, were performed at 10 V and EDX analyses were performed at 15 V. Besides, light microscopy (SteREO Discovery.V20 from ZEISS, Oberkochen, Germany) was also used to obtain the micrographs of the joined samples.

For compositions with 10, 15 and 25% weight of ZnO, additional experiments were performed. The temperature of the pressureless sintering was 750 °C. Instead of using a sandwich structure, the Bi_2_O_3_-ZnO mixtures were applied on the surface of the alumina discs and sintered. The remaining parameters were the same as the above-mentioned experiments. Obtained samples were used to examine the changes occurring during the thermal treatment. Raman spectra of samples were measured (Raman microscope alpha300 R with UHTS300S_VIS laser of wavelength 532 nm from WITec GmbH, Ulm, Germany) and the spectra were obtained using a Zeiss EC Epiplan-Neofluar Dic 100x/0.9 objective from ZEISS, Oberkochen, Germany. These samples were also examined by using an XRD method (X-ray diffractometer apparatus: PANalytical X’Pert Pro MPD with Cu k_α_ from Malvern Panalytical Ltd, UK), where the resulting spectra were analyzed by using X’Pert HighScore Plus software (version 2.0a (2.0.1) produced by PANalytical B.V., Almelo, The Netherlands).

## 3. Results and Discussion

Table 1 presents the different interlayers used, along with the joining temperatures and associated dwell times. Table 1 also informs the reader whether each interlayer was able to successfully bond the ceramic discs under the given conditions. The joined ceramic discs were characterized as either weak bonding or strong bonding depending on an assessment based on the application of low force by hand to try and break the joint. If the ceramic discs fell apart easily under the influence of a low force, such cases are referred to as weak bonding. If the ceramics visually appeared to be satisfactorily joined by the interlayer and also withstood the application of low forces, such cases are referred to as strong bonding.

**Figure 1:**
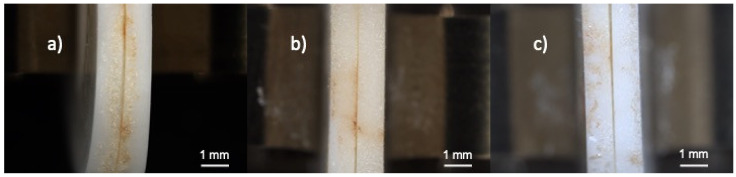
Light microscope images of bonded alumina samples after pressureless treatment in 750 °C with: (**a**) Bi_2_O_3_-5% ZnO interlayer, (**b**) Bi_2_O_3_-10% ZnO interlayer, (**c**) Bi_2_O_3_-15% ZnO interlayer.

The strong joints were obtained for interlayers with 5, 10, 15, 20 and 25% weight of ZnO sintered at 750 °C. The systems after bonding at 750 °C with interlayers consisting of 5, 10 and 15% weight of ZnO are presented in Figure 1. A shiny, yellowish precipitate accumulated on the edges of the samples. The ceramic plates adhere to each other well and there is no visible gap between them.

**Figure 2:**
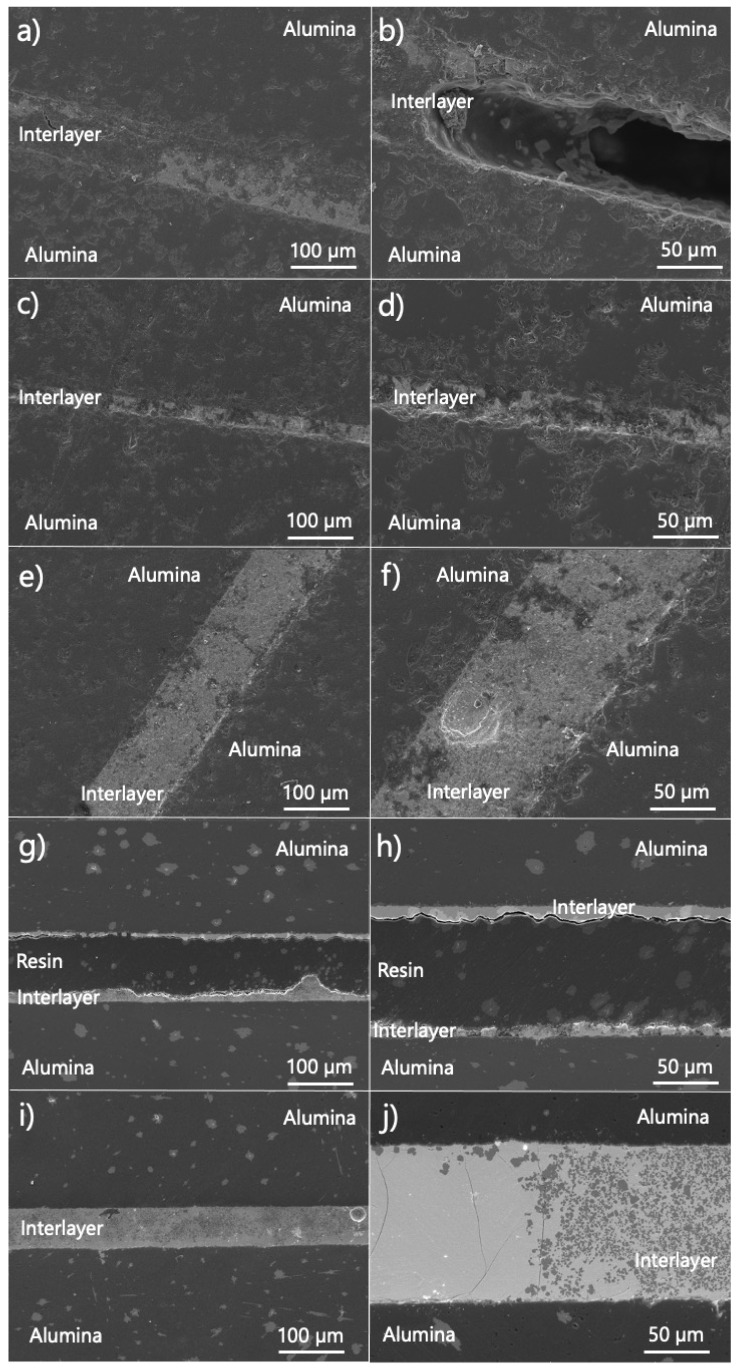
SEM cross-section images of obtained joints after thermal treatment at 750 °C with the different compositions of the interlayers: (**a**,**b**) Bi_2_O_3_-5% of ZnO, (**c**,**d**) Bi_2_O_3_-10% of ZnO, (**e**,**f**) Bi_2_O_3_-15% of ZnO, (**g**,**h**) Bi_2_O_3_-20% of ZnO and (**i**,**j**) Bi_2_O_3_-25% of ZnO.

The cross-section analysis of samples with 5, 10, 15, 20 and 25% weight of zinc oxide was performed by using SEM. The obtained results are presented in Figure 2. As it can be seen, the interlayers with 10, 15 and 25% weight of ZnO have the best quality among the presented figures (Figure 2c–f,i,j). They are uniform and there are no visible holes in the Bi_2_O_3_-ZnO phase. For the composition with 25% weight amount of ZnO (Figure 2j), cracks through the interlayer are noticeable. In the rest of the cross-sections, where the interlayers contain 5 and 20% weight of ZnO, gaps can be noticed (Figure 2a,b,g,h). The lowest integrity of the joint can be assigned to the mixture with 20% weight of ZnO (Figure 2g,h). The system has a lot of free spaces between alumina discs, which were filled with resin during the sample preparation. The interlayer with 5% of ZnO (Figure 2a,b) can be characterized by the appearance of several gaps at different places along the joint. Based on the integrity of the obtained joints, compositions with 5 and 20% weight of ZnO were excluded from the further examinations.

**Figure 3:**
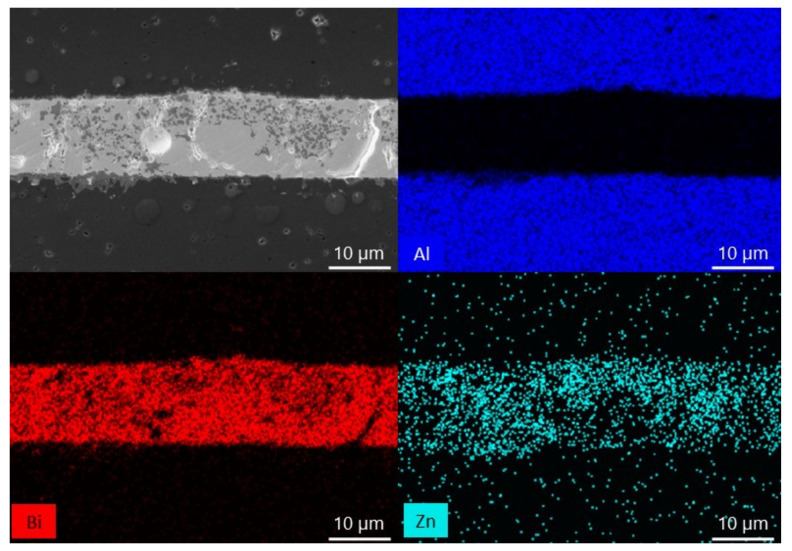
SEM-EDX analysis of the alumina bonded with Bi_2_O_3_-10% ZnO interlayer at 750 °C.

**Figure 4:**
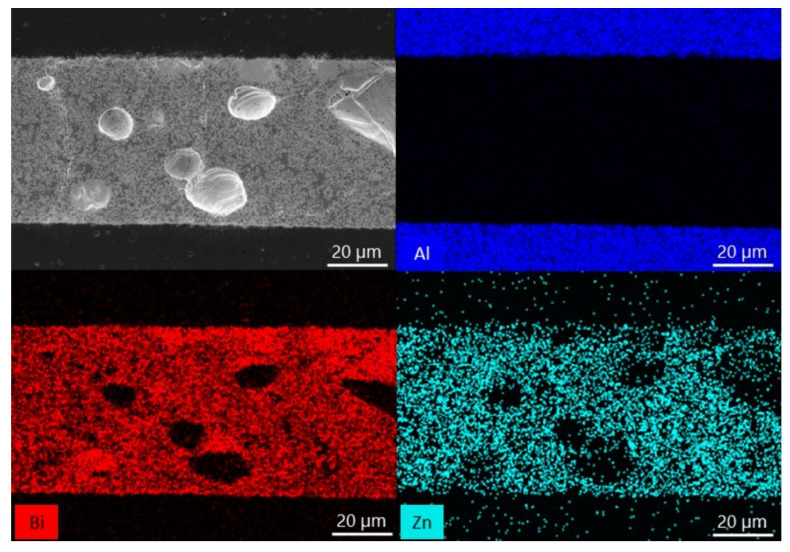
SEM-EDX analysis of the alumina bonded with Bi_2_O_3_-15% ZnO interlayer at 750 °C.

**Figure 5:**
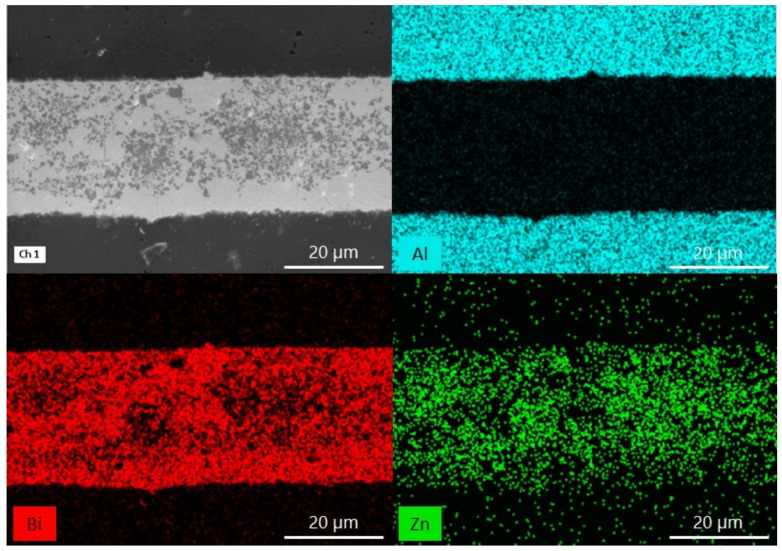
SEM-EDX analysis of the alumina bonded with Bi_2_O_3_-25% ZnO interlayer at 750 °C.

EDX analyses of sample cross-sections with a ZnO weight of 10, 15 and 25% were performed to examine the diffusion mechanism occurring during the bonding (Figure 3, Figure 4 and Figure 5). In each image, two phases can be distinguished in the interlayer: a matrix with a lighter color and a precipitate marked with a darker color. The results show that Zn is the component that is mainly present in the precipitate and Bi is not. It can also be seen that Zn is the component which moved from the interlayer to the ceramic. It seems that the migration of Bi and Al atoms is not evident.

**Figure 6:**
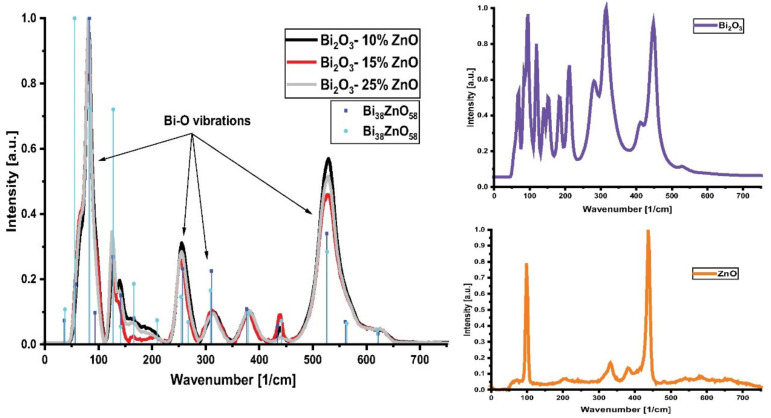
Raman spectra of samples; (**a**) alumina coated with mixtures of Bi_2_O_3_ and ZnO and sintered at 750 °C. The vertical drop lines are the peaks associated with the phase Bi_38_ZnO_58_ [37]; (**b**) the Bi_2_O_3_ raw material used (before sintering); (**c**) the ZnO raw material used (before sintering).

To further investigate the reactions occurring during the experiments, micro-Raman analysis was performed on samples coated with mixtures of Bi_2_O_3_ and 10, 15 and 25% weight of ZnO and sintered at 750 °C. The results are presented in Figure 6a. As can be seen, the obtained spectra are very similar to each other with minor differences in the normalized peak intensities at 167 cm^−1^, 440 cm^−1^, and 529 cm^−1^. The Raman spectra of the raw materials Bi_2_O_3_ and ZnO are also presented in Figure 6b,c respectively. Phase changes have clearly occurred while sintering at 750 °C and, based on the literature, Bi_38_ZnO_58_ could be the reaction product [37].

**Figure 7:**
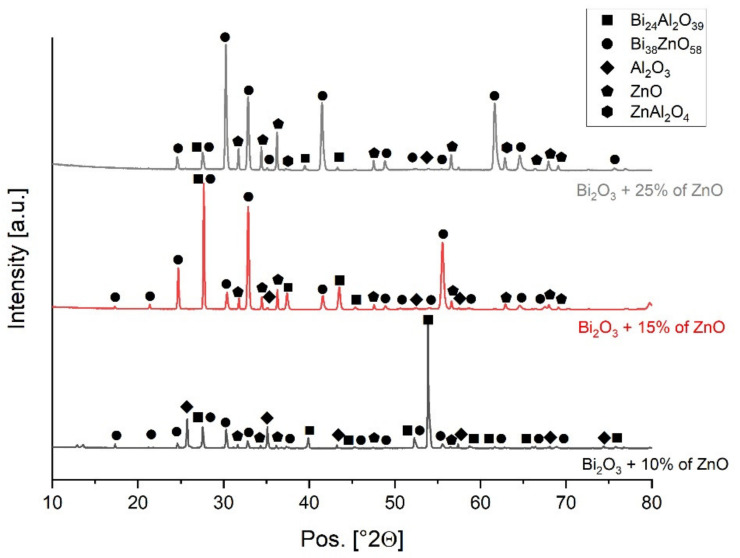
XRD spectra of alumina coated with mixtures of Bi_2_O_3_ and ZnO and fired at 750 °C.

The ceramic surfaces coated with the interlayer and sintered at 750 °C were examined by XRD as well to investigate further the bonding reactions that occur. The peaks were matched to phases using the Highscore plus software (Figure 7). The XRD spectra of Bi_38_ZnO_58_ and Bi_24_Al_2_O_39_ are very similar, with differences mostly in peak intensities at the 2θ values. As a result, some of the peaks were assigned to both phases. In the samples with more ZnO (i.e., samples with 15% and 25% ZnO), the Bi_38_ZnO_58_ peak had the highest intensity. Meanwhile, in the sample with only 10% ZnO, the peak assigned to the Bi_24_Al_2_O_39_ phase had the highest intensity. The XRD pattern also detects the presence of alumina and ZnO. ZnAl_2_O_4_ seems to be scarcely present; however, the presence of this phase might be masked by the predominant peak intensities associated with the Bi_38_ZnO_58_/Bi_24_Al_2_O_39_ phases. Moreover, these spectra were acquired on the surface of the applied interlayer, which was not in contact with alumina. Therefore, it is possible that there was less interaction between the alumina and the part of the interlayer that was analyzed by XRD and Raman. The chemical compositions of the three interlayers after thermal treatment are quite similar to each other. Besides the detected starting materials ZnO, Bi_2_O_3_ and Al_2_O_3_, the results show the presence of Bi_38_ZnO_58_ and Bi_24_Al_2_O_39_. Nevertheless, it is interesting to note that different concentrations of ZnO result in differences in the relative peak intensities of the three interlayers. The spectrum of ceramic coated with Bi_2_O_3_ and 25% weight of ZnO has peaks that can be assigned to ZnAl_2_O_4_.

**Figure 8:**
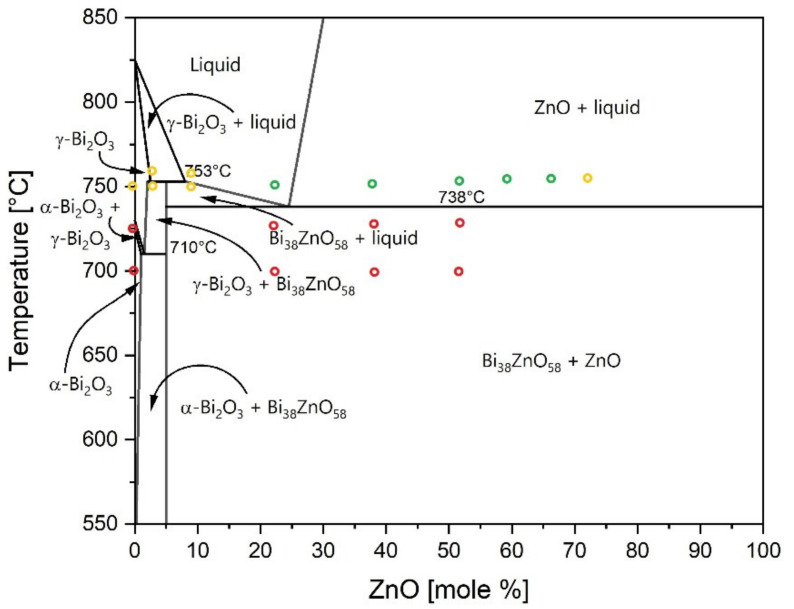
Phase diagram of Bi_2_O_3_-ZnO system; the circles in the phase diagram indicate the tested interlayer compositions and joining temperatures. Red circles indicate no bonding, yellow circles indicate weak bonding and green circles indicate strong bonding (Reprinted/adapted with permission from Ref. [36]. 2004, Springer Nature).

To facilitate the comparison with the phase diagram given in the literature, weight percentage of ZnO was recalculated into the molar percentage [36]. Based on the obtained results, the bonding compositions range can be marked in the phase diagram between Bi_2_O_3_ and ZnO (Figure 8). As it can be seen on the phase diagram (Figure 8), the formation of liquid is necessary to obtain a durable joint. The liquid formation was proven by observed flowing of the melted phase and wetting of the edges of the samples. After the experiments, a shiny precipitate accumulated all around the samples (Figure 1). Without the presence of the liquid, the adherence between components was not achieved. However, the liquid phase does not guarantee a strong bond between alumina. Satisfactory results were not achieved for the composition with a lower amount of ZnO (10.5% molar (2% of weight)). Only higher concentrations of ZnO gave a good joint (with 23.2% molar (5% weight)). Additionally, it can be noticed that the formation of a second phase is an important factor. The phase diagram shows (Figure 8) that the formation of γ-Bi_2_O_3_ and Bi_38_ZnO_58_ results only in weak bonding, while the presence of unreacted ZnO helps to achieve a strong joint. Based on that, the reactivity between ZnO and alumina can be suspected, which influences in a significant way the durability of joints. The only exception was noticed for the interlayer with 30% weight of ZnO. The composition has both unreacted ZnO and a liquid phase at the joining temperature, but only a weak joint was achieved. This suggests that there is probably an upper limit of ZnO content that can inhibit the joining process. This is probably because of the low amount of liquid formation that occurs during heating.

Based on the cross-section analysis of the bond with 20% weight of ZnO (Figure 2g,h), the joints have sufficient strength to hold the ceramics together under the application of a low force, even without a continuous interlayer. Probably, the mixture of Bi_2_O_3_ and ZnO creates a very strong bond with alumina. A discontinuous interlayer suggests that the wettability/adhesion of Bi_2_O_3_-ZnO system on alumina, in that case, is not sufficient. Based on the trend observed in our results (Figure 2), it is possible that the wettability of the interlayer does not have a simple linear relationship with the amount of ZnO introduced in the system. However, it was not systematically checked. It requires a separate study. Additionally, the joint with 5% weight of ZnO was also unsatisfactory (Figure 2a,b). By comparing this result with the phase diagram between components of the interlayer, it can be said that having two phases during heating—liquid and solid—is necessary to obtain a fine joint.

The EDX analysis of the sample cross-sections (Figure 3, Figure 4 and Figure 5) show a matrix and a precipitate formation during the thermal treatment. Based on the obtained results, most likely, the particles can be assigned to ZnO, which did not interact with Bi_2_O_3_ and alumina during the thermal treatment. It proves the presence of two phases at elevated temperature: ZnO and liquid. Moreover, taking also the phase diagram between Bi_2_O_3_ and ZnO under consideration, a conclusion about the chemical composition of the matrix can be made: it is likely that bismuth zinc oxide, Bi_38_ZnO_58_, is formed [36]. Besides, it can be said that Zn is a diffusing component in the system. However, previous works with the use of just Bi_2_O_3_ as the interlayer showed that Bi is the component which diffuses [29]. It means that by adding another substance, which changes the chemical composition, the bonding mechanism also changes. This can be explained based on the dimensions of the atom. Bi has a rather significant atomic radius (160 pm). Zn, with a smaller atomic radius (135 pm), can move more easily between the crystal lattice of alumina [38]. The ability of the diffusion of ZnO into Al_2_O_3_ lattice was proven before, during a solid-state reaction between the components resulting in spinel formation [39]. Moreover, Bi atoms are probably more involved in forming Bi_38_ZnO_58_ and Bi_24_Al_2_O_39_.

The similarity between the Raman spectra of samples coated with mixtures of Bi_2_O_3_ and 10, 15 and 25% weight of ZnO (Figure 6) suggests that during the thermal treatment similar processes have taken place in every applied interlayer. Moreover, almost every peak can be matched to the ones coming from the literature spectra of sillenite-type crystals such as Bi_38_ZnO_58_ [37]. Bi_38_ZnO_58_ is a possible reaction product that forms during the bonding process. The positions of the obtained peaks, along with the literature values, are shown in Table 2. It proves the hypothesis from EDX analysis that this compound is one of the phases that forms during the joining of the alumina ceramic. In the Raman spectra, the most intensive peaks are from the Bi-O vibrations occurring in every system. As a result, the peaks of Bi_24_Al_2_O_39_ could be masked. The Raman spectra could also cover the peaks coming from e.g., spinel formation, which is expected to occur at a lower rate.

Based on XRD results (Figure 7), it can be assumed that the occurring reactions are similar in all examined coated samples. By interaction with Bi_2_O_3_, ZnO formed the expected compound, Bi_38_ZnO_58_, which proves the previous conclusion. Additionally, the spectrum of coating with 25% weight of Zn also shows the presence of spinel ZnAl_2_O_4_, which is a product of the reaction between ZnO and alumina ceramic. It proves, that by adding ZnO into the system, the interaction between this compound and alumina can occur and influence the bonding strength. The lack of ZnAl_2_O_4_ peaks in the other two spectra can be caused by a smaller amount of spinel. The ZnO content is lower, which can influence the product formation. However, as it can be seen, not all of ZnO bonded with Bi_2_O_3_ and alumina but still can be clearly found in the bonding system. Hence, applying longer dwell time can decrease the amount of unreacted zinc oxide after the thermal treatment and increase the bonding strength. The presence of the bismuth aluminum oxide shows that not only ZnO is the reactive component, but that Bi_2_O_3_ also reacts with alumina, which was noticed during previous bonding experiments with this compound [29]. The metastable phase Bi_24_Al_2_O_39_ forms, which was proven to be a product of the reaction between Bi_2_O_3_ and Al_2_O_3_ [31]. The reactions occurring during the bonding process can be summarized with equations presented below:

$$19\;{\mathrm{Bi}}_{2}O_{3}+\;\mathrm{ZnO}\;\to \;{\mathrm{Bi}}_{38}{\mathrm{ZnO}}_{58}$$

$$\mathrm{ZnO}+\;{\mathrm{Al}}_{2}O_{3}\;\to \;{\mathrm{ZnAl}}_{2}O_{4}$$

$$12\;{\mathrm{Bi}}_{2}O_{3}+\;{\mathrm{Al}}_{2}O_{3}\;\to \;{\mathrm{Bi}}_{24}{\mathrm{Al}}_{2}O_{39}$$

## 4. Conclusions

In this work, bonding between alumina was achieved by applying mixtures of Bi_2_O_3_ and ZnO as interlayers. The bonding was achieved by transient liquid phase bonding and by pressureless thermal treatment at a relatively low temperature of 750 °C. Based on the phase diagram between Bi_2_O_3_ and ZnO, the temperature can be probably decreased to 738 °C, which is the liquid formation temperature in the examined system.

Mixtures of Bi_2_O_3_ with 5, 10, 15, 20 and 25% weight of zinc oxide gave bonding results between alumina ceramics. The bonding ability is strictly connected with the liquid formation during the thermal treatment. However, the presence of unreacted Bi_2_O_3_ and ZnO influences the bonding properties of the mixtures. By adding a sufficient amount of ZnO, the obtained joints are durable and have satisfactory integrity. The quality of the joint was best in the case of 10, 15 and 25% weight of ZnO.

In the system, not only Bi_2_O_3_ reacts with the alumina ceramic, as proven in previous research. ZnO interacts with alumina as well, forming ZnAl_2_O_4_ spinel. Most probably, this reaction increases the strength of the bonds. Moreover, Zn is the component, which diffuses into the ceramic.

Further research should focus on optimizing the thermal process. It was proven that not all ZnO reacts, and therefore, increasing time of the thermal treatment can influence the strength and the stability of joints. Additionally, the interlayer structure might influence the bonding process. Most likely, different results will be obtained by applying the components layer by layer. Besides, the influence of Bi_38_ZnO_58_ on the interlayers and bonding is unknown. It is possible that replacing Bi_2_O_3_ with a different oxide that does not react with ZnO can increase the spinel formation.

## Figures and Tables

**Table 1:** Details and results of conducted joining experiments.

Interlayer	Temperature [°C]	Dwell Time [h]	Result
Bi_2_O_3_	700	2	No bonding
Bi_2_O_3_-5% ZnO	700	2	No bonding
Bi_2_O_3_-10% ZnO	700	2	No bonding
Bi_2_O_3_-15% ZnO	700	2	No bonding
Bi_2_O_3_	725	3	No bonding
Bi_2_O_3_-5% ZnO	725	3	No bonding
Bi_2_O_3_-10% ZnO	725	3	No bonding
Bi_2_O_3_-15% ZnO	725	3	No bonding
Bi_2_O_3_	750	3	Weak bonding
Bi_2_O_3_-0.5% ZnO	750	3	Weak bonding
Bi_2_O_3_-2% ZnO	750	3	Weak bonding
Bi_2_O_3_-5% ZnO	750	3	Strong bonding *
Bi_2_O_3_-10% ZnO	750	3	Strong bonding *
Bi_2_O_3_-15% ZnO	750	3	Strong bonding *
Bi_2_O_3_-20% ZnO	750	3	Strong bonding *
Bi_2_O_3_-25% ZnO	750	3	Strong bonding *
Bi_2_O_3_-30% ZnO	750	3	Weak bonding
Bi_2_O_3_-0.5% ZnO	760	3	Weak bonding
Bi_2_O_3_-2% ZnO	760	3	Weak bonding

* Interlayers were selected for further investigation.

**Table 2:** Peak positions from Raman spectra of coated alumina samples with the literature values.

Compositions with Bi_2_O_3_ and:			Literature Spectra of Bi_38_ZnO_58_ [37] [cm^−1^]
10% of ZnO [cm^−1^]	15% of ZnO [cm^−1^]	25% of ZnO [cm^−1^]	
			56.4
81	81	81	83.0
			93.2
125	125	125	126.7
141	141	141	141.3
167	167	167	166.0
203	203	203	209.2
255	255	255	256.8
314	314	314	310.7
381	381	381	376.5
440	440	440	434.2
529	529	529	526.1
631	631	631	621.5

## Data Availability

Data is contained within the article. Raw untreated Raman and XRD spectra are available on request from the corresponding author.
